# Large-Scale Association Study Confirms Genetic Complexity Underlying Type 2 Diabetes

**DOI:** 10.1371/journal.pbio.0000030

**Published:** 2003-10-13

A leading cause of death and disability, diabetes affects some 16 million Americans and up to 135 million people worldwide. While environmental factors such as diet and lifestyle are known to influence an individual's risk of getting adult-onset, or Type 2, diabetes, there is also a substantial inherited component, though many of the genetic pathways involved remain unidentified.

The challenge of defining these genetic pathways lies in the fact that diabetes is what is known as a “complex trait”: not only is it likely that variations in many different genes or some combination of genes contribute to an increased risk, but there are probably different genes associated with diabetes in different populations. Tackling the monumental task of deciphering this genetic puzzle in the largest known study of its kind, Inês Barroso and colleagues confirm the genetic complexity of the disease and clearly demonstrate that untangling the genetics will require even larger studies.

In diabetes, defects in both the secretion and function of insulin—which is produced by the pancreas—impair the body's ability to metabolize glucose. Based on what is known about the biology of pancreatic function and diabetes, the researchers chose 71 potential suspect genes that could reasonably be expected to contribute to the disease if they malfunctioned. Some of these genes are involved in the function of pancreatic beta-cells, which secrete insulin; a second group influences the function of insulin and glucose metabolism; and a third plays a broader role in energy metabolism. The results show that none of the genes on their own had a large effect, though a number of gene variations increased risk slightly. They also suggest the existence of variations in several genes that influence the risk of Type 2 diabetes.

The dataset will be valuable for future studies of diabetes and supports the view that variation in genes affecting insulin production as well as insulin action can influence the risk of Type 2 diabetes. However, the genetic complexity of the disease—with a number of genes conveying a slightly increased risk— demands additional studies of larger populations to reliably identify the genes involved and the genetic variations that, alone or in combination, increase or lower an individual's risk of developing the disease.

